# Exogenous and Endogenous Determinants of Blood Trihalomethane Levels after Showering

**DOI:** 10.1289/ehp.10049

**Published:** 2007-10-11

**Authors:** Lorraine C. Backer, Qing Lan, Benjamin C. Blount, J.R. Nuckols, Robert Branch, Christopher W. Lyu, Stephanie M. Kieszak, Marielle C. Brinkman, Sydney M. Gordon, W. Dana Flanders, Marjorie Romkes, Kenneth P. Cantor

**Affiliations:** 1 National Center for Environmental Health, Centers for Disease Control and Prevention, Chamblee, Georgia, USA; 2 Division of Cancer Epidemiology and Genetics, National Cancer Institute, Rockville, Maryland, USA; 3 Department of Environmental & Radiological Health Sciences, Colorado State University, Fort Collins, Colorado, USA; 4 Center for Clinical Pharmacology, University of Pittsburgh, Pittsburgh, Pennsylvania, USA; 5 Battelle/Centers for Public Health Research and Evaluation, Durham, North Carolina, USA; 6 Battelle, Columbus, Ohio, USA

**Keywords:** CYP2D6, CYP2E1, disinfection by-products, drinking water disinfection, GSTT1, showering exposures, trihalomethanes

## Abstract

**Background:**

We previously conducted a study to assess whether household exposures to tap water increased an individual’s internal dose of trihalomethanes (THMs). Increases in blood THM levels among subjects who showered or bathed were variable, with increased levels tending to cluster in two groups.

**Objectives:**

Our goal was to assess the importance of personal characteristics, previous exposures, genetic polymorphisms, and environmental exposures in determining THM concentrations in blood after showering.

**Methods:**

One hundred study participants completed a health symptom questionnaire, a 48-hr food and water consumption diary, and took a 10-min shower in a controlled setting. We examined THM levels in blood samples collected at baseline and 10 and 30 min after the shower. We assessed the significance of personal characteristics, previous exposures to THMs, and specific gene polymorphisms in predicting postshower blood THM concentrations.

**Results:**

We did not observe the clustering of blood THM concentrations observed in our earlier study. We found that environmental THM concentrations were important predictors of blood THM concentrations immediately after showering. For example, the chloroform concentration in the shower stall air was the most important predictor of blood chloroform levels 10 min after the shower (*p* < 0.001). Personal characteristics, previous exposures to THMs, and specific polymorphisms in *CYP2D6* and *GSTT1* genes were significant predictors of both baseline and postshowering blood THM concentrations as well as of changes in THM concentrations associated with showering.

**Conclusion:**

The inclusion of information about individual physiologic characteristics and environmental measurements would be valuable in future studies to assess human health effects from exposures to THMs in tap water.

Epidemiologic studies have consistently found significant associations between exposure to drinking water disinfection byproducts (DBPs) and human cancers (Cantor et al. 1998; Freedman et al. 1997; Hildesheim et al 1998; King and Marrett 1996; McGeehin et al. 1993; Villanueva et al. 2006b; Zierler et al. 1988); however, the reported odds ratios were typically < 2.0 and quite variable. Studies of adverse reproductive outcomes associated with DBP exposures have yielded inconsistent results (e.g., King et al. 2000; Savitz et al. 1995, 2006; Waller et al. 1998). As suggested by Villanueva et al. (2006a), additional information, including individual physiologic and genetic characteristics, recent relevant exposures, and direct environmental measurements, may improve exposure assessment and thus the precision of disease risk estimates associated with human exposure to disinfection byproducts.

We previously conducted a study to assess whether, in addition to drinking water, household water uses that generate aerosols or release volatile chemicals are an important and measurable source of trihalomethane (THM) exposure (Backer et al. 2000). Increases in blood THM levels among the 10 subjects who showered or bathed were highly variable and fell into two significantly different groups, suggesting possible differences in THM metabolism. For example, the mean (± SD) increase in blood bromoform levels was 8.2 ± 2.97 pg/mL for one group of five participants and 21.2 ± 4.26 pg/mL for the remaining five participants. Miles et al. (2002) reported finding similar clustering of blood THM levels after various household water use activities.

The variation of blood THM levels may have been the result of differences in physiologic characteristics or recent related exposures. For example, THM blood levels depend not only on tap water quality, but also on the nature and frequency of water use activities (Nuckols et al. 2005).

Blood levels of THMs after environmental exposures may reflect individual differences in drug metabolizing enzyme gene polymorphisms. For example, brominated THMs are substrates for glutathione *S*-transferase theta (GSTT)–mediated glutathione conjugation reactions (Landi et al. 1999). Individuals with the active enzyme may clear brominated THMs from the blood more rapidly than similarly exposed individuals with the null genotype, and thus have lower levels (Landi et al. 1999).

Polymorphisms in metabolic genes may also mediate disease risks. *GSTT1* polymorphisms may be important in determining an individual’s risk for non-Hodgkin lymphoma, stomach cancer, and liver cancer associated with exposure to dichloromethane (Kerridge et al. 2002; Lan et al. 2001; Setiawan et al. 2000). The cytochrome P450 gene *CYP2D6* (cytochrome P450 2D6) is involved in metabolism of xenobiotic chemicals (Nebert and Russell 2002). It has 5–10 variants affecting metabolic activity to influence levels of parent compound and metabolites that may be responsible for adverse health effects. For *CYP2E1* (cytochrome P450 2E1), Infante-Rivard (2004) found that exposure to high levels of THMs affected fetal growth only in individuals with a specific genetic polymorphism. In addition, specific variants of *CYP2E1* could be involved in the metabolic activation of trihalomethane-related carcinogens (Gao and Zhang 1999).

The primary objective of this study was to determine the major contributors to variability in blood THM levels after showering, and particularly to evaluate whether the clustering of blood THM levels after showering that we observed in our previous study was a spurious finding because of our small sample size or was real based on physiologic and genetic differences among individuals.

## Materials and Methods

### Institutional review board approvals

The institutional review boards of the Centers for Disease Control and Prevention (CDC), the National Institutes of Health, the General Clinical Research Center (GCRC) at the University of Pittsburgh, and Battelle Memorial Institute approved this study protocol. We have complied with all applicable requirements for protection of human subjects. Study participants gave written informed consent before the study.

### Study participants

This study was conducted at the GCRC clinical research laboratory during July–September 2004. We recruited potential study participants from a panel who volunteered to participate in research at the GCRC. Study participants provided blood samples for a complete blood count, standard blood chemistry panel, and enzyme activity and genotyping, including *CYP2D6* (accession no. AY545216; GenBank; http://www.ncbi.nlm.nih.gov/GenBank), *CYP2E1* (accession no. DQ515958; GenBank)*, GSTT1* [glutathione *S*-transferase (theta class); accession no. BC007065; GenBank], and *GSTM1* [glutathione *S*-transferase (μ class); accession no. BC024005; GenBank]. Eligible subjects (18–45 years of age) had a normal blood screen, were not pregnant (verified for women with a pregnancy test on the day of the study), not nursing an infant, and physically able to take a shower and provide blood samples. All subjects were nonsmokers, did not drink an average of more than one alcoholic beverage per day, were willing to abstain from drinking alcoholic beverages for 2 days before the study, did not take acetaminophen five or more times a week, and were free from chronic lung disease and asthma.

Estimates of the frequency of *GSTT1* positive in the various populations range from 15 to 80% (El-Masri et al.1999; Garte et al. 2001; Raimondi et al. 2006). Therefore, we used the initial GCRC genotyping results to identify potential participants who were *GSTT1* positive. We recruited 43 study participants with *GSTT1* positive (*GSTT1* wild type or *GSTT1* heterozygous null) and 57 with *GSTT1* homozygous null genotypes. We then assessed these individuals for their *CYP2D6* and *CYP2E1* genotypes.

Of 112 volunteers, 100 met eligibility criteria, agreed to participate, and completed study activities over a 6-week period. A random sample of nine subjects repeated the study for analysis of the repeatability of our results (analysis not shown here). There were five family groups, and 11 subjects were blood relatives of another study participant.

### Questionnaire and diary

We collected questionnaire data for each participant just before conducting study activities. Specifically, we collected data on demographics (age, sex, race), height, weight, occupation, smoking, use of chloral hydrate, recent respiratory symptoms, and activities that were sources of THM exposure (bathing, swimming, using hot tub or sauna, washing clothes or dishes) or that might affect the activity of the enzymes of interest [alcohol consumption (Dupont et al. 2000), using solvents or cleaners containing volatile organic compounds (VOCs)].

Study participants recorded in a diary how much tap water they drank during the 48 hr preceding study activities. We estimated cold water intake as number of 8-oz cups. Weisel and Chen (1994) found that heating water increased THM concentrations and that household exposures calculated using the THM concentration in heated water were 50% higher than those calculated using the THM concentration in cold water. Thus, we estimated hot water intake as 2 × [number of bowls of soup made with water + 8-oz cups of hot coffee + 8-oz cups of hot tea + (servings of hot cereal × 0.66) + (servings of rice or pasta × 0.66) + (servings of vegetables cooked in water × 0.05)].

Certain foods induce synthesis of some of the metabolic enzymes of interest in this study. Subjects recorded in a diary how many servings of broccoli, cauliflower, lettuce, onions, garlic, watercress, and black tea they ate/drank during the 48 hr preceding study activities (Kall et al.1996; Leclercq et al. 1998; Le Marchand et al. 1999; Stupans et al. 2001). We estimated the intake of foods of interest as the number of servings of broccoli, cauliflower, and lettuce + (servings of onions × 0.2) + (servings of garlic, watercress, and black tea × 0.1).

### Study activities

To minimize the influence of foods, beverages, alcohol consumption, and selected medications on study results, participants fasted overnight, did not drink any alcoholic beverages or take medications that affect the enzymes of interest (i.e., dextromethorphan, chlorpheniramine, chloral hydrate) during the 48 hr before study activities, and did not drink caffeinated beverages on the day of the study. Urine samples were collected immediately before and after study activities for future analysis of haloacetic acid concentrations and one extra blood sample was collected for future genotyping.

On the day of the study, subjects completed the questionnaire and provided two 10-mL blood samples, took a 10-min temperature-controlled shower, provided one 10-mL blood sample first 10 min and then 30 min after turning off the shower, and then ingested a 250-mg chlorzoxazone tablet (Lemmon Company, Sellersville, PA). One 10-mL blood sample was drawn 2 hr later for analysis of chlorzoxazone metabolism as a measure of CYP2E1 enzyme activity. Study participants remained at the study site for an additional 2 hr to ensure that no adverse reaction to chlorzoxazone occurred.

To limit study participants’ exposure to THMs unrelated to the shower, we required that they not flush the toilet or run tap water while in the study area, dry off and dress as quickly as possible, and stay in a separate room away from further exposure to THMs. We provided study participants with THM-free bottled drinking water.

### Biological specimens

Whole blood samples for THM analysis were collected by a certified phlebotomist using Vacutainer tubes processed to remove VOC contamination (Cardinali et al. 1995). Samples were analyzed for THM levels using headspace solid phase microextraction (SPME) coupled with gas chromatography (GC) and high-resolution mass spectrometry (MS). Analyte quantification was based on stable isotope dilution (Bonin et al. 2005).

### In vivo CYP2E1 *activity.*

*CYP2E1* is inducible but the genotype does not predict the phenotype. We therefore measured CYP2E1 activity by the *in vivo* chlorzoxazone test (Frye et al. 1998; Scott et al. 1999). Serum concentrations of chlorzoxazone and its major metabolite, 6-hydroxychlorzoxazone, were measured by high performance liquid chromatography. We used the ratio of 6-hydroxychlorzoxazone to chlorzoxazone as a phenotypic measure of CYP2E1 activity.

### GSTT1*,* CYP2D6, *and* CYP2E1 *genotyping.*

Genomic DNA was extracted from peripheral blood using the PureGene DNA Isolation Kit (Gentra Systems, Inc., Minneapolis, MN) according to the manufacturer’s instructions. We screened the CYP2D6*3 and *4* and CYP2E1*5 variant alleles with TaqMan allele discrimination-based assays using the Applied Biosystems 7700 system (Applied Biosystems, Foster City, CA). For the differential polymerase chain reaction (PCR) method for *GSTT1* genotyping, we used a housekeeping gene (β-globin) as an internal control (Pemble et al. 1994).

Positive and negative PCR controls were included with each amplification reaction. For both genotyping analyses, we used previously sequenced genomic DNA samples as positive controls for the homozygous wild-type, heterozygous, and homozygous variant genotypes with every PCR analysis to verify reproducibility of the assay and to confirm accuracy of genotype classifications. Approximately 10% of randomly selected samples were repeated blindly for verification of genotyping assays. All results were interpreted independently by two laboratory staff members, and no discordant genotype classifications were identified.

### Environmental samples

#### Air

We collected three air samples near the subject’s breathing zone: a preexposure bathroom sample, a 10-min time-integrated sample in the enclosed shower stall during the shower, and a 5-min time-integrated postshower sample in the unventilated bathroom. Air samples were collected using evacuated stainless steel canisters. Filled canisters were sealed and shipped to Battelle (Columbus, OH) for analysis. We analyzed the samples for THMs by automated GC/MS using a modified version of U.S. Environmental Protection Agency method TO-14 (Winberry et al. 1990). Full details of air sampling and analysis procedures are available elsewhere (Gordon et al. 2006).

#### Water samples

The shower head was modified to allow remote water sampling. Duplicate samples were collected 5 min after each shower began. Participants were instructed to set the shower water temperature between 104 and 105°F (40–41°C). Shower water temperature was monitored by the participant and study staff via a digital thermometer in the shower stall and a remote radio thermometer outside the bathroom. Water samples were collected in borosilicate glass vials containing sodium thiosulfate to quench further THM formation and phosphate buffer to standardize pH between 6.0 and 6.5. We analyzed water samples for THM levels using headspace SPME–GC/MS with quantification based on stable isotope-dilution (Cardinali et al. 2004).

### Statistical analyses

We conducted statistical analyses using SAS, version 9.0 (SAS Institute Inc., Cary, NC). We evaluated associations of predictor variables (e.g., demographics, genotype, THM concentrations in air, THM concentrations in water) with THM levels in blood 10 min after showering and THM level changes (10 min minus baseline) after showering. For changes in THM levels in blood, the logs of the differences (10 min minus baseline) were used as our outcome measure. The residuals from the final models were evaluated for normality; this assumption was satisfied for each model.

We used generalized estimating equations to model these data to account for the correlations caused by having multiple members of the same family in the analyses. Univariate analyses were run separately for each THM. We chose independent variables that had a *p*-value ≤ 0.10 for inclusion in initial multivariate models and used a backward selection approach to select a final model for each outcome measure. After choosing a final model for the change in blood levels of THMs at 10 min after exposure, we wished to investigate the contribution of individual variables to the total variability explained by all variables. The partial *r*^2^ measures the marginal contribution of one explanatory variable when all others are already included in the model. Partial *r*^2^ values were not available in the generalized estimating equations procedure, so our *r*^2^ results were based on a least-squares analysis.

## Results

In this article, we present the results from the analysis of environmental samples and blood samples collected at baseline and at 10 min after the shower.

Approximately half (54) of our 100 study participants were women, and most (73) were white. Eighty-five study participants reported eating at least one of the foods of interest within 24 hr of the study, and about one-fifth reported having upper respiratory symptoms within 4 weeks of the study. Eleven subjects reported exposure to bleach, and five or fewer people reported exposures to specific solvents or other VOCs within 24 hr of the study (data not shown). Over half (57) of our study participants were *GSTT1* null; 43 were *GSTT1* positive (*GSTT1* wild type or *GSTT1* heterozygous null). Nearly three-fourths (74) of our study participants were *CYP2D6* wild type (*1/*1), 21 were heterozygous (*1/*3 or *1/*4), pand 5 were homozygous recessive (*4/*4). The results of the chlorzoxazone assay for CYP2E1 enzyme activity were as follows: median, 0.54; interquartile range, 0.37–0.72; range, 0.21–1.72. Consistent with previous reports (Gurley et al 2002; Lucas et al 1998), the distribution of 6-hydroxychlorzoxazone/chlorzoxazone ratios was skewed toward lower values (i.e., lower metabolic activity).

Results from the analyses of THMs in shower water and shower stall air samples are presented in Table 1. Chloroform was present in the highest concentrations in both water and air samples, followed by concentrations of bromodichloromethane, dibromochloromethane, and bromoform. We prevented air circulation during study activities; thus, THM air concentrations in the 10-min integrated shower samples and the 5-min integrated postshower samples were similar.

**Figure 1:**
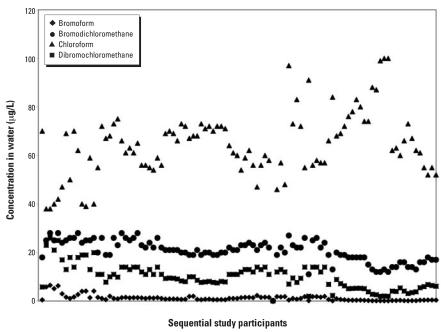
THM concentrations in shower water. Data are presented by study ID number in numeric and chronologic order over the 12-week study period (*n* = 99).

The distribution of THMs in water samples by consecutive study participant number over the 12-week study period is shown in Figure 1. Bromoform levels were > 6 μg/L for all but one of the first 12 participants and < 2 μg/L for the remaining participants. The drop in bromoform concentration during the study was likely the result of unusually high rainfall that occurred in Pittsburgh at the time of the study. It is likely that the unusually high water volume diluted the local water source, thus diluting tap-water bromine concentrations. Despite the very low levels of bromoform in the water, we were able to detect bromoform in 75 of the baseline blood samples and in 98 of the 10-min postshowering blood samples. The concentrations of mono-and dibrominated compounds also decreased over the study period. Over the study period, chloroform levels in shower water varied from < 40 μg/L to nearly 100 μg/L.

**Figure 2:**
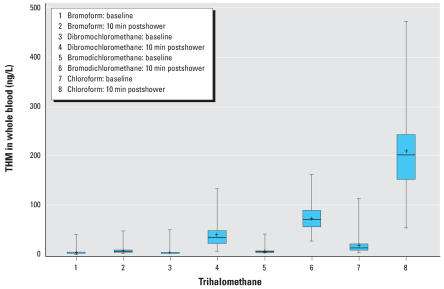
THM concentrations in whole blood at baseline and 10 min postshowering. The capped bars are the minimum and maximum, the box extents indicate the 25th and 75th percentiles, the line inside the box marks the 50th percentile, and the + indicates the mean (*n* = 99).

THM concentrations in blood samples and the changes in concentration (10 min postshower minus baseline) are presented in Table 2. The blood THM data for one study participant seemed anomalous (> 2 SDs from the sample means at baseline and after showering) and thus were removed from these analyses. Blood THM levels are presented in Figure 2. THM concentrations measured 10 min after the shower were log-normally distributed.

**Figure 3:**
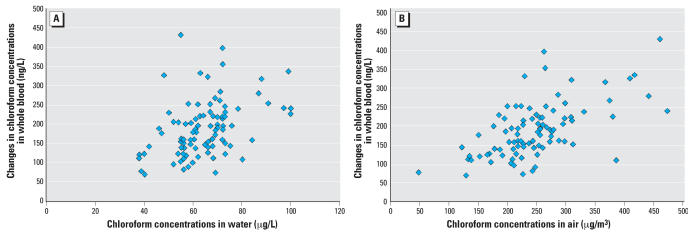
Associations between the change in chloroform concentrations in whole blood (10 min postshower minus baseline) and THM concentrations in shower water (*A*) and the 10-min integrated shower air sample (*B*). The correlation (*r*^2^) between changes in chloroform concentrations in whole blood and in shower water was 0.60 (*p* < 0.001). The correlation between chloroform concentrations in water and the 10-min integrated shower air sample was 0.63 (*p* < 0.001) (*n* = 99).

The magnitude of postshower increases in blood THMs reflected THM concentrations in shower water and air. The data for chloroform are presented in Figure 3. The blood concentrations of bromoform reflected water concentrations of bromoform.

Variables that were statistically significant (*p* ≤ 0.10) in univariate analyses were included in initial models for THM levels in blood. We included water and air (10-min integrated air sample) THM levels; height; weight; body mass index (BMI); sex; race; age; alcohol consumption; and recent swimming, sauna use, and water-related household activities (doing laundry, washing dishes). We also included genotype (for *CYP2D6* and *GSTT1*), and the ratio of 6-hydroxychlorzoxazone to chlorzoxazone (for *CYP2E1*) in our models. A summary of statistics from GENMOD procedure (SAS Institute Inc.) modeling blood levels of THMs 10 min postshowering and the change in THM concentrations (10 min postshower minus baseline) is presented in Table 3.

Ten minutes after the shower, shower water concentration was an important predictor of individual blood levels of all the THMs except chloroform. For bromodichloromethane and chloroform, which are more volatile than the other THMs, air concentrations were important in determining blood levels.

Previous exposures and personal characteristics also affected blood THM levels at 10 min postshowering. Swimming was the most important predictor of blood bromoform levels, and higher BMI was associated with lower blood levels of all THMs except chloroform. *CYP2D6* genotypes with decreased metabolizing activity were significant predictors of increased blood bromodichloromethane and chloroform levels, and the *GSTT1* null (inactive enzyme) genotype was associated with an increase in chloroform blood levels.

The important predictor variables in the models for the changes (10 min postshower minus baseline) in blood THM concentrations were similar to those that were important in predicting 10-min postshower blood THM concentrations (Table 3). For example, the CYP2D6 enzyme variant with decreased metabolizing activity was associated with higher 10-min postshower blood concentrations of bromodichloromethane and chloroform and with higher postshower changes in blood concentrations of bromodichloromethane and dibromochloromethane. Similar to the 10-min postshower concentrations, the *GSTT1* null genotype was associated with greater changes in chloroform concentrations 10 min postshower. Environmental concentrations, hot water intake, and BMI were important in determining the postshower changes in blood levels of all THMs.

The importance of the environmental concentrations of THMs in determining the increase in blood levels was confirmed when we examined the partial *r*^2^. For chloroform and bromoform, the partial *r*^2^ for variables in the final model included 0.3841 (*p* < 0.001) for log(chloroform concentration in air) and 0.3434 (*p* < 0.0001) for log(bromodichloromethane concentration in air), respectively. *r*^2^ for the other variables in these models were smaller by an order of magnitude. For bromoform and dibromochloromethane, the partial *r*^2^ for variables in the final model included 0.86 (*p* < 0.0001) for log(bromoform concentration in water) and 0.80 (*p* < 0.0001) for log(dibromochloromethane concentration in water), respectively. Again, *r*^2^ for other variables in the model were smaller by at least an order of magnitude.

## Discussion

In this study, we found that environmental concentrations (water, air) were the most important predictors for increases in blood THM levels from a showering exposure. We also found that personal characteristics, previous exposures, and metabolic enzyme polymorphisms were significant modulators of shower-related increases in blood concentrations for some THMs.

In assessing the effects of personal characteristics on internal levels of an environmental contaminant, it is important not only to carefully measure exposure, but also to limit exposure variability. In the present study, we limited experimental dose variability by controlling the duration and temperature of the shower. We also enhanced exposure measurements by including concentrations of THMs in shower air as well as water in our analyses. We did not see the clustering of blood THM concentrations that we observed in our previous study (Backer et al. 2000), suggesting that the earlier results were likely a spurious effect attributed to a small (*n* = 10) sample size. However, consistent with the earlier study, we found an association between shower water and postshowering blood THM concentrations. In the previous study, the average chloroform concentration in shower water was 31 μg/L and the average change in blood concentration (10 min post-shower minus baseline) was approximately 90 ng/L. Here, the geometric mean chloroform concentration in shower water was approximately 64 μg/L, and the average change in blood concentration (10 min postshower minus baseline) was approximately 190 ng/L. Our results demonstrate that blood chloroform and other THM levels are related to tap-water levels in a dose–response manner and that showering is an important source of THM exposure that should not be overlooked in conducting studies of disease risk.

Once exposure to exogenous chemicals occurs, genetic variability in metabolic enzymes may play a role in determining the relative internal dose of parent compound and metabolites. In our previous study (Backer et al. 2000), we noted that THM concentrations in blood samples collected 10 min after a shower appeared to cluster in two groups. In this study, we did not replicate this observation; however, we found that enzyme gene polymorphisms were significant predictors for levels of the most highly chlorinated THMs in blood 10 min after the shower. When compared with the wild type, the reduced activity genotypes *CYP2D6* (*4/*4) or *GSTT1* null were associated with increased chloroform (and thus presumably decreased metabolites) in the blood 10 min after exposure. Although not significant, the heterozygous extensive metabolizing *CYP2D6* (*1/*4) genotype suppressed the magnitude of the increases in chloroform and bromodichloromethane in the blood 10 min after exposure. The phenotypic variation in *CYP2E1* did not affect blood THM levels in our study. Our results provide support for the idea that individual genotypic variation can modulate systemic exposure, and thus health risks, associated with exposure to THMs by affecting the relative concentrations of parent THMs and detoxified or activated metabolites present in an individual’s blood.

We were particularly interested in the effects of genetic polymorphisms on bromoform metabolism. Our choice of study site was based on historical data indicating we could expect tap-water levels of all THMs to be high enough to be detectable in blood after a 10-min shower. However, as noted, tap-water bromoform concentrations decreased to concentrations < 2 μg/L after the first 12 subjects. Consequently, blood levels of bromoform 10 min postshowering were consistently low (often below detection) and not highly variable, and we were unable to address the effects of genetic polymorphisms on bromoform levels in blood.

Although evaluating individual genotypes is likely to be useful in interpreting the results of epidemiologic studies of environmental exposures, it requires collecting a biologic specimen from each study participant, thus increasing the resources needed for a given study. To enrich our study populations with the genotypes of interest without making the sample size prohibitively large, we prescreened study participants for specific genotypes. Even with this enrichment, we were able to consistently detect differential effects associated with enzyme polymorphisms only for chloroform.

Overall, our study results indicate that the most important predictors of blood THM concentrations after showering are the environmental exposures themselves. This is particularly evident in the decrease in blood bromoform levels that paralleled the decrease in water levels over the study period. Using the geometric mean concentration of bromoform as the reference, the ratios of bromodichloromethane and dibromochloromethane were similar in the shower water (20.1 and 8.5, respectively) and in the air during the shower (25.2 and 9.2, respectively). Chloroform is the most volatile of the THMs, and the ratio of chloroform to bromoform was of the same magnitude but higher in shower air (99.7) than in shower water (63.5). Again using the geometric mean concentration of bromoform as a reference, the ratios of bromodichloromethane and chloroform were very similar when comparing the levels in blood 10 min after the shower. Thus, immediately after showering, the relative amounts of the different THMs were consistent with what was in shower water and air.

Environmental exposure levels in both water and air were also significant predictors of the changes in blood THMs between baseline and 10 min postshowering. This is consistent with reports by Xu and Weisel (2005a, 2005b) that uptake of chloroform during showering occurs through both dermal and inhalation exposure routes, respectively.

Increased BMI was a predictor for smaller changes in blood THM levels. Compared with subjects with lower BMIs, subjects with higher BMIs tended to have lower blood THM levels at 10 min postshowering; this association may be attributed to THMs partitioning into lipid (Batterman et al. 2002). These results suggest that, given a specific exposure and a baseline measurement, personal characteristics such as BMI can modulate the distribution of these lipophilic DBPs within the body.

Of interest is the association of swimming with blood levels of bromoform and chloroform. Swimming within 48 hr of study activities was the most important predictor of blood bromoform levels 10 min after exposure. This may be because the bromoform levels were very low in our study showers compared with levels present in swimming pool water and in poolside air (Fantuzze et al. 2001). Because bromoform is a carcinogen (National Toxicology Program 1989), studies assessing health risks from THM exposures should query subjects about chlorinated pool use for a more complete assessment of brominated THM exposure. From our data, it is not clear why swimming would have a negative association with the postshower change in blood chloroform concentration. This association disappears with adjustment for baseline values; however, the pros and cons of such adjustment are not always clear (Glymour et al. 2005).

In summary, our analyses indicate that individual polymorphisms in *GSTT1* and *CYP2D6* genes were significant but minor predictors of blood trihalomethane levels 10 min after showering and of the differences between blood levels 10 min after showering and those at baseline. We demonstrated that it is important to quantify environmental contaminant levels when defining dermal and inhalation exposure to THMs. We also found that personal characteristics, such as BMI, and recent exposures, such as swimming and drinking hot tap water–based beverages, affected postshowering blood THM levels. Our study suggests that information about individual susceptibility factors, such as enzyme gene polymorphisms, together with information about individual physiologic characteristics and environmental measurements, should be included as a component of exposure assessment in future studies of human health risks from exposure to THMs.

## Figures and Tables

**Table 1:** THM concentrations [median (interquartile range)]^a^ in environmental samples: tap water collected during showering and three integrated air samples (preexposure baseline, a time-integrated sample in the shower stall while the participant is showering, and a time-integrated sample to cover the 5-min postshower exposure period).

		Air (μg/m^b^)		
THM^c^,^d^	Water (μg/L)	Preexposure baseline sample	10-min integrated shower stall sample	5-min integrated post-shower bathroom sample
Bromoform	1.0 (0.4–1.5)	< LOD^e^	2.69 (1.46–4.13)	2.69 (1.46–4.34)
Dibromochloromethane	9.5 (6.2–13)	< LOD	27.6 (20.5–38.8)	29.5 (20.4–38.6)
Bromodichloromethane	21 (18–24)	< LOD	75.0 (59.1–86.2)	78.7 (64.1–87.8)
Chloroform	66 (56–72)	1.31 (0.69–1.61)	245 (212–279)	248 (221–288)
Total THMs	98 (91–102)	1.31 (0.69–1.61)	353 (314–394)	362 (328–408)

LOD, limit of detection.

a Range is the 25th and 75th percentiles.

b LOD for THM concentrations in air: bromoform, 2.1 μg/m^3^; dibromochloromethane, 1.7 μg/m^3^; bromodichloromethane, 1.3 μg/m^3^; chloroform, 0.98 μg/m^3^.

c LOD for trihalomethanes in water: bromoform: 0.12 μg/L; dibromochloromethane: 0.24 μg/L; bromodichloromethane: 0.48 μg/L; chloroform: 0.92 μg/L.

d When the concentration of an analyte was below the LOD, the concentration was replaced with the LOD/√2.

e Except for chloroform, the THM concentrations in air were below the LOD. Only chloroform was included in the total THM value for air.

**Table 2:** THM concentrations (ng/L) in blood samples collected from 99 study participants immediately before showering and blood samples collected from the same participants 10 min after completing the shower, and the changes in THM concentrations (10 min postshower minus baseline).

	Median concentration (interquartile range^a^)		Median difference (interquartile range)
THM^b^	Baseline postshower	10 min minus baseline	10 min postshower
Bromoform	0.91 (0.7–1.2)	4.0 (2.2–6.2)	2.9 (1.5–4.5)
Dibromochloromethane	1.2 (0.71–2.1)	32 (21–46)	31 (20–42)
Bromodichloromethane	2.2 (1.4–3.5)	69 (54–88)	64 (49–84)
Chloroform	10 (6.7–18)	200 (150–240)	187 (144–230)

a Range is the 25th–75th percentiles.

b Limit of detection (LOD) for THMs in blood: bromoform: 0.55 ng/L; dibromochloromethane: 0.23 ng/L; bromodichloromethane: 0.24 ng/L; chloroform: 2.4 ng/L. When the concentration of an analyte was below the LOD, the concentration was replaced with LOD/√2.

**Table 3:** Summary of statistics from the multivariate generalized linear models procedure modeling the log of blood levels of THMs at 10 min postshower and the log of the differences in blood THMs (10 min post-shower minus baseline).

Trihalomethane	Parameter	Estimate	95% CIs
10 min postshower			
Bromoform	Intercept	1.21#	0.77 to 1.64
	BMI^a^	−0.02**	−0.04 to −0.01
	Swam^b^	1.43#	1.29 to 1.56
	Water concentration^c^	0.56#	0.46 to 0.65
Dibromochloromethane	Intercept	2.63#	2.28 to 2.99
	BMI	−0.02**	−0.03 to −0.01
	Water concentration	0.08#	0.06 to 0.11
	Air concentration^d^	0.01**	0.003 to 0.02
Bromodichloromethane	Intercept	3.18#	2.84 to 3.52
	BMI	−0.01*	−0.02 to −0.003
	Sauna^e^	0.14*	0.02 to 0.25
	Hot water intake	−0.002#	−0.003 to −0.001
	CYP2D6 *1/*4^f^	−0.11	−0.26 to 0.04
	CYP2D6 *4/*4	0.20*	0.005 to 0.39
	Water concentration	0.03*	0.01 to 0.05
	Air concentration	0.01*	0.001 to 0.01
Chloroform	Intercept	4.58#	4.37 to 4.80
	Chlorine cleaners^g^	−0.15*	−0.27 to −0.02
	Hot water intake	−0.002#	−0.003 to −0.001
	CYP2D6 *1/*4	−0.08	−0.20 to 0.04
	CYP2D6 *4/*4	0.31**	0.08 to 0.54
	*GSTT1* null^h^	0.10	0.00 to 0.20
	Air concentration	0.003#	0.002 to 0.004
10 min postshower minus baseline			
Bromoform	Intercept	1.91#	1.47 to 2.36
	BMI	−0.03**	−0.05 to −0.01
	Hot water intake	−0.003#	−0.004 to −0.002
	Log water concentration^i^	1.01#	0.92 to 1.10
Dibromochloromethane	Intercept	1.62#	1.23 to 1.98
	BMI	−0.01**	−0.02 to 0.005
	Hot water intake	−0.003#	−0.004 to −0.002
	CYP2D6 *1/*4	−0.11	−0.25 to 0.03
	CYP2D6 *4/*4	0.23*	0.06 to 0.40
	Air concentration	0.01*	0.001 to 0.013
	Log water concentration	0.88#	0.70 to 1.07
Bromodichloromethane	Intercept	1.21*	0.001 to 2.41
	BMI	−0.01*	−0.02 to −0.002
	Hot water intake	−0.003#	−0.004 to −0.002
	CYP2D6 *1/*4	−0.10	−0.25 to 0.05
	CYP2D6 *4/*4	0.2*	0.005 to 0.40
	Log air concentration^j^	0.76#	0.47 to 1.06
Chloroform	Intercept	1.21	0.22 to 2.20
	BMI	−0.01**	−0.02 to −0.004
	Swam	−0.13*	−0.24 to −0.01
	Hot water intake	−0.003#	−0.004 to −0.002
	*GSTT1* null	0.13*	0.02 to 0.24
	Log air concentration	0.43**	0.12 to 0.75
	Log water concentration	0.45*	0.03 to 0.86

Values represent the convergence of the algorithm developed with GENMOD (SAS Institute Inc.) and are parameter estimates and 95% confidence intervals (CIs) of variables with *p* < 0.05. *n* = 99.

a Calculated from height and weight data (CDC 2005).

b Swam in a chlorinated pool within 48 hr of doing study activities.

c Concentration of the analyte in water (μg/L).

d Concentration of analyte in air (μg/m^3^).

e Used sauna within 48 hr of doing study activities.

f Genotype groups: *1/*1 = wild type is the comparison group; *1/*4 and *1/*3 = heterozygous extensive metabolizers (should have high ratio); *4/*4, *1/*5,*5B/*5B (also referred to as *5/*5) = genetic variants with significantly decreased metabolizing activity (should have low ratio).

g Participant used household bleach or cleaners with bleach within 48 hr of doing study activities.

h GSTT1 null, compared to GSTT1 positive.

i Log [concentration of analyte in water (μg/L)].

j Log [concentration of analyte in air (μg/m^3^)].

* *p* < 0.05.

** *p* < 0.01.

# *p* < 0.001.
